# From Clones to Buds and Branches: The Use of Lung Organoids to Model Branching Morphogenesis *Ex Vivo*

**DOI:** 10.3389/fcell.2021.631579

**Published:** 2021-03-04

**Authors:** Ana Ivonne Vazquez-Armendariz, Susanne Herold

**Affiliations:** ^1^Department of Internal Medicine II, Cardio-Pulmonary Institute, Universities of Giessen and Marburg Lung Center, Giessen, Germany; ^2^German Center for Lung Research, Giessen, Germany; ^3^Institute for Lung Health, Giessen, Germany

**Keywords:** branching morphogenesis, lung development, stem cells, lung organoids, cell-fate decisions

## Abstract

Three-dimensional (3D) organoid culture systems have rapidly emerged as powerful tools to study organ development and disease. The lung is a complex and highly specialized organ that comprises more than 40 cell types that offer several region-specific roles. During organogenesis, the lung goes through sequential and morphologically distinctive stages to assume its mature form, both structurally and functionally. As branching takes place, multipotent epithelial progenitors at the distal tips of the growing/bifurcating epithelial tubes progressively become lineage-restricted, giving rise to more differentiated and specialized cell types. Although many cellular and molecular mechanisms leading to branching morphogenesis have been explored, deeper understanding of biological processes governing cell-fate decisions and lung patterning is still needed. Given that these distinct processes cannot be easily analyzed *in vivo*, 3D culture systems have become a valuable platform to study organogenesis *in vitro*. This minireview focuses on the current lung organoid systems that recapitulate developmental events occurring before and during branching morphogenesis. In addition, we also discuss their limitations and future directions.

## Introduction

Organoids are 3D structures derived from stem cells that proliferate and give rise to organ-specific cells types capable to form structures that recapitulate the cellular architecture and functions of the native organ. Organoids can be regenerated from embryonic stem (ES) cells, induced pluripotent stem cells (iPSC) and adult stem cells (Lancaster and Knoblich, 2014). Human and murine lung organogenesis consist of five overlapping phases with progressive branching generation: embryonic, pseudoglandular, canalicular, saccular, and alveolar (Wells and Melton, 2000). Regarding human ES cells, studies have shown that stem cells obtained from the pseudoglandular or canalicular stage during lung development are an efficient source of stem cells with the potential to differentiate into airway and alveolar cell lineages (Mondrinos et al., 2014; Rosen et al., 2015). Conversely, iPSC are derived from adult somatic cells reprogrammed by addition of time-specific growth factors that mimic the main known molecular cues necessary for lung organogenesis (Takahashi et al., 2007; Morrisey and Hogan, 2010). The most effective protocols to redirect murine and human pluripotent stem cells (hPSC) generate at first, definitive endoderm followed by anterior foregut endoderm (AFE), ventral anterior foregut endoderm (VAFE) generation and, lastly, differentiation into NKX2.1^+^ lung progenitors (Huang et al., 2015). Depending on the culture conditions, there are now several lung organoid systems capable to some extent resemble the cellular and structural complexity of the bronchioalveolar compartment of the lung (Table 1) (Rawlins et al., 2009; Dye et al., 2015, 2016; Aurora and Spence, 2016; Hawkins et al., 2017; McCauley et al., 2017, 2018; Nikolić et al., 2017; Miller et al., 2019).

**Table 1:** Murine and human lung organoid models mimicking features of lung development *in vitro*.

**Lung organoid** **system**	**Epithelial cell components**	**Main organoid-forming cell type**	**Supporting cell type**	**References**
Tracheospheres	Basal, ciliated and goblet cells	Human and murine basal cells	-	Rock et al., 2009
iPSC-derived alveolar organoids	AECI and AECII	NKX2.1^+^ CPM^+^VAFE lung progenitors	HLF (culture possible but not required)	Gotoh et al., 2014; Yamamoto et al., 2017
iPSC-derived alveolar organoids	AECII	NKX2.1^+^ CD47^hi^CD36^low^ lung progenitors	MF	Hawkins et al., 2017
Human airway-like organoids	Basal, club, ciliated and goblet cells	NKX2.1^+^FOXA2^+^ CPM^+^ lung progenitors	-	Konishi et al., 2016
Fetal bud tip-derived organoids	Basal, ciliated and goblet cells or AECI and AECII	Human epithelial tips	HLF	Nikolić et al., 2017
iPSC-derived airway organoids	Basal, ciliated and club cells or alveolar progenitors	NKX2.1^+^ FOXA2^+^VAFE lung progenitors	-	McCauley et al., 2017
HLO	Basal, club and ciliated cells; AECI and AECII	NKX2.1^+^ FOXA2^+^ CPM^+^ VAFE lung progenitors	hPSC-derived fibroblasts (VIM^+^ αSMA^+^PDGFRα^+/−^ mesenchyme)	Dye et al., 2015, 2016
LBO	Goblet cells; AECII	NKX2.1^+^ FOXA2^+^ CPM^+^ lung progenitors	hPSC-derived fibroblasts (CD90^+^VIM^+^ mesenchyme ≤ 2%)	Chen et al., 2017
PLO	Club and goblet cells; AECII	NKX2.1^+^ FOXA2^+^CPM^+^ lung progenitors	-	Miller et al., 2018
Bronchioalveolar organoids	Club, ciliated, and goblet cells; AECI and AECII	BASC	LuMEC	Lee et al., 2014
BALO	Basal, club, ciliated and goblet cells; AECI and AECII	BASC	rMC	Vazquez-Armendariz et al., 2020

*iPSC, induced pluripotent stem; CPM, Carboxypeptidase M; VAFE, ventral anterior foregut endoderm; FOXA2, Forkhead-Box-Protein A2; HLF, human lung fibroblasts; AECI, alveolar epithelial cells type 1; AECII, alveolar epithelial cells type 2; MF, mouse fibroblasts; HLO, human lung organoids; hPSC, human pluripotent stem cells; PDGFRα, platelet-derived growth factor receptor A; αSMA, alpha smooth muscle actin; LBO, lung bud organoids; VIM, vimentin; PLO, patterned lung organoids; BASC, bronchioalveolar stem cells; LuMEC, primary mouse lung endothelial cells; BALO, bronchioalveolar lung organoids; rMC, resident mesenchymal cells*.

Lung development in mice begins in the AFE from where the two primary lung buds develop at embryonic day 9.5 (~28th day in men). These buds comprise three cellular layers: an inner epithelial layer enclosed by mesenchyme and a thin external mesothelial layer (Morrisey and Hogan, 2010; Rawlins, 2011). Notably, branching morphogenesis is mediated by signals coming from the surrounding mesenchyme, including cues implicated in regulation of early embryonic cell-fate decisions, such as fibroblast growth factors (FGFs), epidermal growth factor, transforming growth factor (TGF)-β, WNT, hedgehog (HH), retinoic acid (RA), and NOTCH (Shannon et al., 1998; Wells and Melton, 2000). In this review, we will describe the current lung organoids platforms that model distinct stages of lung morphogenesis. We will also discuss the limitations of these models, and how such systems could be improved to more closely reflect lung development and provide deeper insights into molecular and cellular mechanisms involved in branching morphogenesis.

## Organoid Systems Modeling Cell-Fate Decisions and Patterning of the Lung

During the embryonic phase, the endoderm undergoes sequential cell-fate decisions giving rise to lung progenitor cells with higher lineage restriction. In this regard, epithelial progenitor cell appearance dependents on the specific region and cell type. For instance, restricted expression of the transcription factor NKX2.1 at the VAFE is an early marker for lung specification, marking the region where the trachea and primary lung buds would appear (Morrisey and Hogan, 2010). In line with these findings, several lung organoid models have been generated using iPSCs. 3D co-culture of hPSC-derived carboxypeptidase M (CPM)-expressing cells with human lung fibroblasts supplemented with alveolar-associated growth factors, formed alveolar epithelial organospheres comprising NKX2.1 and CPM-expressing cells, as well as mature AQP5^+^ alveolar epithelial cells type 1 (AECI) and SFTPC^+^ alveolar epithelial cells type 2 (AECII) (Gotoh et al., 2014). Of note, higher induction of SFTPC^+^ AECII was achieved by addition of fetal lung fibroblasts into the cultures (Yamamoto et al., 2017). Given that CPM expression overlapped with NKX2.1, CPM was then identified as a cell-surface marker for VAFE. Accordingly, CPM^+^ progenitor cells gave rise to airway-like organoids with ciliated and mucus-producing cells when the culture medium was supplemented with a NOTCH inhibitor (Konishi et al., 2016). In another study, cell sorting of labeled NKX2.1- AQP5^+^ alveolar epithelial cells type 1 (AECI) and SFTPC^+^ alveolar epithelial cells type 2 (AECII) obtained from hPSCs developed into organoids expressing EpCAM and SFTPC when co-cultured with mouse fibroblasts (Hawkins et al., 2017). Notably, sorted NKX2.1-GFP^+^ cells validated CPM as a progenitor cell marker and revealed a novel cell surface phenotype, CD47^hi^CD36^low^, for NKX2.1-expressing lung progenitor cells. Nevertheless, these organoid models gave rise to either airway or distal epithelial cell lineages but did not undergo proximal-to-distal differentiation.

During the pseudoglandular stage, transcription factor SOX2 expression defines proximal epithelial lung progenitors that will later differentiate into neuroendocrine, secretory and ciliated cells. Conversely, distal progenitors located at the murine bud tips of the expanding tubes are characterized by SOX9 and ID2 expression (Rawlins et al., 2009). Yet, recent studies using *in vitro* human-derived cultures, including micro-dissected embryonic bud tip-derived organoids have demonstrated low levels of SOX2 in the distal human lung (Kim et al., 2016; Nikolić et al., 2017). Similarly, McCauley and colleagues reported a hPSC-derived lung bud tip organoid model using NKX2-1^+^ progenitors that could give rise to either proximal airway or alveolar lineages (McCauley et al., 2017). In this organoid model, blockage of WNT signaling activation decreased SOX9 and increased SOX2 expression, leading to appearance of proximal airway organoids containing secretory, ciliated, and basal cells, whereas preservation of WNT signaling promoted development of distal epithelial organoids composed solely of alveolar progenitors. While these organoid models could, at some extent, mimic lung epithelial patterning, these organoids were unable to undergo further elongation, branch formation or alveolarization.

Although most of the lung organoids derived from adult stem cells lack proximal to distal patterning, these models have been proven useful for studying cell-fate decisions happening at later stages of branching morphogenesis. For example, murine lung organoids termed tracheospheres are generated from isolated P63^+^NGFR^+^ basal cells and consist of a P63^+^KRT5^+^KRT14^+^NGFR^+^ basal cell layer surrounded by an additional laminal layer of fully differentiated KRT8^+^ ciliated and goblet cells (Rock et al., 2009). Following treatment with BMP signaling pathway inhibitors, murine tracheospheres displayed increased basal cell proliferation and higher colony formation capacity, however, basal cell differentiation into ciliated or goblet cells was significantly impaired (Mou et al., 2016; Tadokoro et al., 2016). Alternatively, human basal cells have been shown to form tracheospheres, bronchospheres or nasospheres depending on whether the basal cells are derived from the trachea, large airways or nasal epithelium, respectively. Human tracheospheres also include basal, ciliated and goblet cells (Danahay et al., 2015; Butler et al., 2016; Hild and Jaffe, 2016). Based on *in vivo* experiments, NOTCH signaling was proposed as a relevant regulator of secretory and ciliated cell differentiation (Rock et al., 2011). In this regard, screening of 5,000 compounds in human bronchospheres confirmed these data and also identified NOTCH signaling pathway as a mediator of basal cell differentiation. Blockage of NOTCH1 receptor caused upregulation of basal cell markers, while inhibition of NOTCH2 receptor led to a significant increase in ciliated cell markers expression but not goblet cells markers (Danahay et al., 2015). Additionally, CRISPR/Cas9 genome editing of human basal cells followed by organoid culture, Gao and colleagues identified the transcription factor grainyhead-like 2 (Grhl2) as a molecular regulator of barrier function and ciliated cell differentiation (Gao et al., 2015). Notably, loss of Grhl2 inhibited organoid growth and reduced the expression of NOTCH and ciliogenesis genes with known Grhl2 regulatory sites. Going forward, combination of CRISPR/Cas9 technology with such organoid systems could represent a powerful tool to promptly screen for genes modulating epithelial cell-fate decisions occurring late during branching formation.

Even though the mentioned *in vitro* organoid systems cannot recapitulate developmental environmental cues and cellular interactions necessary to fully drive cell-fate decisions or lung patterning during branch formation, a mixture of *in vivo* approaches and organoid models can certainly be utilized to strengthen cellular and molecular characterization of crucial events taking place at the distinct stages of human lung branching morphogenesis.

## Branching Lung Organoid Models

**Figure 1:**
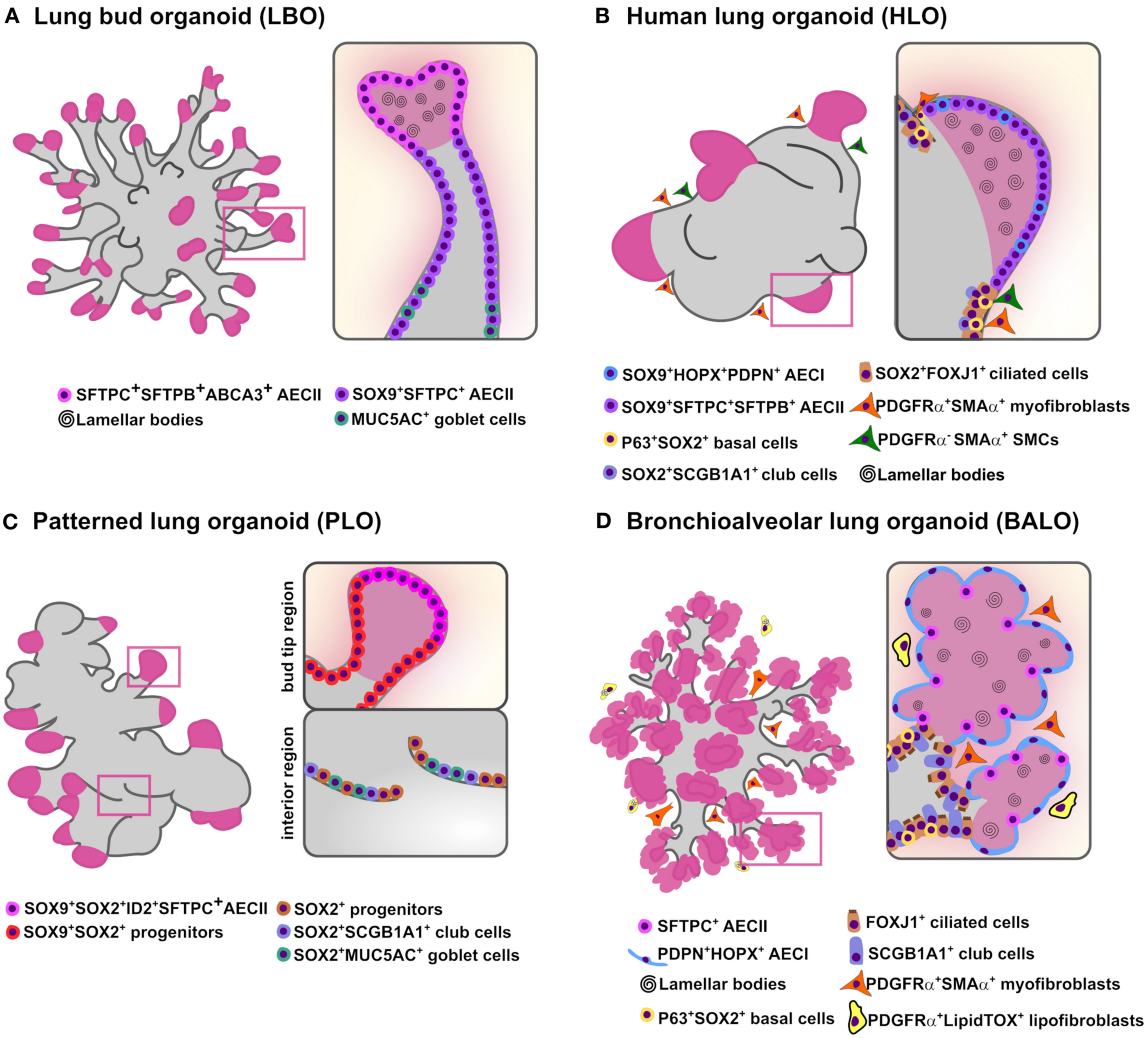
*In vitro* branching lung organoid models. Schematic representation of the current lung organoids models showing proximodistal specification and bud/branching formation *in vitro* derived from hPSCs and murine adult stem cells. **(A)** Lung bud organoids (LBO) are derived from hPSCs and have several cells expressing AECII markers SFTPC, SFTPB, and ABCA3 while airway goblet cell markers MUC5AC and MUC5AB are rare. While most cells co-expressed SOX9^+^ the pheripheral luminal cells expressed only mature AECII markers (MUC1, SPTPC, SFTPB, ABCA3) containing lamellar bodies **(B)** Human lung organoids (HLO) are derived from hPSCs and display proximal airways, expressing proximal cell type-specific markers for basal cells (P63), ciliated cells (FOXJ1) and club cells (SCGB1A1) co-expressing SOX2; distal-like structures expressing SFTPC/SFTPB AECII and PDPN/HOPX AECI cell-type markers mostly co-stained with SOX9. Lamellar bodies are found inside and outside the SFTPC^+^ AECII-like cells while SMA^+^ PDGFRα^+^ (myofibroblasts) and SMA^+^ PDGFRα^−^ [smooth muscle cells (SMCs)] are observed in close proximity to the epithelial tube-like structures. **(C)** Patterned lung organoid (PLO) are derived from hPSCs and have peripheral regions that contain cells co-expressing SOX9/SOX2, some also SFTPC and ID2 while internal regions only express SOX2 with some cells expressing the club cells marker SCGB1A1 and goblet cells marker MUC5AC. **(D)** Bronchioalveolar lung organoids (BALO) are derived from BASC isolated from the lung of adult mice and possess proximal tubular structures containing club/secretory cells (SCGB1A1), basal cells (P63), ciliated cells (β-4 tubulin) and distal alveolar-like structures are composed of AECII (SFTPC) and AECI (PDPN). Lamellar bodies are observed in AECII and in the lumen. Myofibroblasts (PDGFRα^high^αSMA^+^) are observed close to BALO's branching points while lipofibroblasts (PDGFRα^low^LipidTOX^+^) are observed in close proximity to AECII.

During the pseudoglandular stage of lung development, lung buds go through numerous series of extension and ramification creating a complex tree-like structure (Metzger et al., 2008). Accordingly, Chen and colleagues generated the so-called lung bud organoids (LBOs) from human ES cells and iPSC-derived mesoderm and pulmonary endoderm. In this model, LBOs developed after induction of a ventral lung fate from AFE in the presence of BMP4, FGF10, FGF7, RA, and CHIR99201(GSK3b inhibitor/WNT activator) and resembled the cellular composition of the lung bud tips *in vivo*. After xenotransplantation under the kidney capsule of immunodeficient mice, LBOs gave rise to branching airway (basal, club, ciliated and goblet cells) and early alveolar structures (AECII and immature AECI) displaying proximodistal specification (Chen et al., 2017). Notably, even though progenitor cells cultivated in media containing BMP4, FGF10, FGF7, RA, and CHIR9920 did not give rise to mature airway cells or AECI, LBOs formed well-defined branching structures expressing airway markers (MUC5AC and SCGB3A2) in the proximal structures and AECII markers (HT2-280, MUC1, SFTPC, and SFTPB) in the distal tips (Figure 1A). Cross-referencing with human data, RNA-sequencing analysis of LBOs indicates that these organoids reflect the transcriptomic profile corresponding to the second trimester of human gestation. Moreover, in another study, Dye et al. provided a protocol for hPSC differentiation using a high dose of FGF10 to obtain lung organoids comprising both epithelial and mesenchymal cell lineages (Figure 1B) (Dye et al., 2015). In this study, HH signaling pathway was activated to stimulate NKX2.1 expression which allowed the development of complex epithelial structures called human lung organoids (HLO). HLOs formed proximal airway-like epithelial tubules containing immature airway cell types co-expressing SOX2 (basal, club and ciliated cells), enclosed by mesenchyme (myofibroblast and smooth muscle cells), and possessed distal-like structures comprised by SFTPC^+^ and HOPX^+^ cells co-expressing SOX9. However, a clear branching pattern was not observed in HLOs. To address this issue, in a follow up study, HLOs were first cultured in a bioartificial microporous poly(lactide-co-glycolide) (PLG) scaffold niche and then transplanted into the fat pad of immunocompromised mice (Dye et al., 2016). Transplanted HLOs engrafted and differentiated into an organized pseudostratified airway-like epithelium with a complete repertoire of airway cell types (basal, ciliated, goblet and club cells) and supporting mesenchyme. Yet, this approach did not support AEC survival, implying the need for more cell-specific protocols to drive lung branching specification. In another study, Miller and colleagues determined that addition of FGF7, CHIR-99021 and all-trans RA was required for the formation of patterned lung organoids (PLO) from isolated human fetal bud tips *in vitro* (Figure 1C). In addition, hPSC-derived foregut spheroid organoids cultured under the same conditions developed PLO with an airway-like (SOX2^+^, MUC5AC^+^, and SCGB1A1^+^ cells) and bud tip-like domains (SOX9^+^, SFTPC^+^, and ID2^+^ cells) (Miller et al., 2018). While these models represent compelling tools for visualization of certain aspects of lung formation, the next challenge for such lung branching/patterned organoid systems will be to generate well-defined branched structures with alveolar compartments. That would circumvent the need for organoid transplantation into host mice to drive airway cell maturation.

The canalicular phase of lung development is delineated by further branching, elongation and broadening of alveolar airspaces (Burri, 1984). Particularly, capillaries start to organize around the distal airspaces where the interaction with the neighboring alveolar epithelial cells leads to epithelial cells flattening and subsequent formation of the initial air-blood barriers (Rawlins et al., 2007). Lee and colleagues characterized a 3D co-culture model of murine bronchioalveolar stem cells (BASCs) and a heterogeneous population of lung endothelial cells. In this study, the endothelial-derived BMP4-NFATc1-thrombospondin-1 (TSP1) signaling axis was shown to be important for BASC proliferation and alveolar differentiation (Lee et al., 2014). Notably, complex bronchioalveolar organoids generated club, ciliated and goblet cells and AECII but did not drive full AECI differentiation. At a lesser extent, comparable mixed organoids formed when SCGB1A1^+^ cells were co-cultured with Lgr5^+^ and Lrg6^+^ mesenchymal cells (Lee et al., 2017) or when distal epithelial progenitors (EpCAM^high^itgα6β4^+^CD24^low^) were co-cultured with primary mesenchymal cells (Bertoncello and McQualter, 2011). BASC contribution to lung regeneration upon different types of injury was recently confirmed by lineage tracing of SFTPC^+^SCGB1A1^+^ BASCs in double reporter transgenic mice *in vivo* (Liu et al., 2019; Salwig et al., 2019). In an additional study, 3D culture of BASCs (EpCAM^high^SCA-1^+^SCGB1A1^+^SFTPC^+^) and lung resident mesenchymal cells (rMC) (CD45^−^CD31^−^EpCAM^−^ SCA-1^+^) isolated from adult mouse lungs gave rise to mainly bronchioalveolar lung organoids (BALO) that exhibit distinct bronchiolar-like structures containing basal, club, goblet, and ciliated cells and alveolar-like structures comprising differentiated AECI and AECII that resembled the proximodistal pattering of the lung (Figure 1D) (Vazquez-Armendariz et al., 2020). The BALO system contains distinct subsets of rMCs, including myofibroblasts (PDGFRα^high^αSMA^+^) and lipofibroblasts (PDGFRα^low^LipidTOX^+^) that were shown to be indispensable for proximodistal specification, differentiation, and branching formation. Accordingly, this model could be used to study epithelial-mesenchymal crosstalk mechanisms and the role of these subsets within the niche during lung branching. In addition, following treatment of BALO with morpholino oligos against *miR-142-3p*, a miRNA known to activate the canonical WNT β-catenin pathway, BALO mimicked developmental defects observed in *ex vivo* embryonic lung implants (Carraro et al., 2014; Vazquez-Armendariz et al., 2020). Such defects included reduced growth and impaired branching morphogenesis caused by adenomatosis polyposis coli (*Apc*) and β-catenin destruction complex activation. Of note, BALO model could be supplemented with tissue-resident alveolar macrophages by direct cell microinjection adding another level of complexity to the system and opening new avenues to study the interactions between immune cells and various cellular components *ex vivo*. A significant drawback of BASC-derived organoid models is that BASCs have not been identified in humans; therefore, equivalent human organoid models are still missing. Nevertheless, further BASC genome profiling may reveal specific cell markers that might enable the identification of a human counterpart.

## Next-Generation Lung Organoids

Within the last few years, significant progress has been made on organoid technology; nevertheless, major research challenges remain to be addressed in order to rely on 3D organoid platforms to study lung developmental biology. For instance, current human lung organoid models lack terminal cellular maturity and structural complexity, particularly, maturation of the alveolar lineages and progressive formation of branches in hPSC-derived organoids. Nevertheless, there are diverse directions that could be taken to achieve these key features of branching morphogenesis.

First, cell microinjection of relevant cell types into lung organoids to monitor the cellular interactions happening during branching formation. In this regard, lipofibroblasts are a type of lung-resident mesenchymal cell that are found in close proximity to AECII and have been shown to be involved in mesenchymal-epithelial crosstalk during alveolarization (Rehan and Torday, 2012; Barkauskas et al., 2013). Therefore, given that the use of direct cell microinjection into organoids for analyzing cellular interactions has been demonstrated (Vazquez-Armendariz et al., 2020), AECII maturation and differentiation within the “branching” lung organoids could potentially be achieved by addition of supporting cells that would provide the necessary mediators to drive branching formation and alveolarization. Notably, complex hPSC-derived lung organoids contain supporting mesenchyme (Dye et al., 2016; Chen et al., 2017). However, full characterization of the mesenchymal populations found within these models has not been performed. For instance, direct introduction of distinct supporting cells (e.g., myofibroblast, lipofibroblasts, fetal macrophages) to the developing organoids at specific time-points by microinjection might help to determine how and when distinct supporting cells contribute to alveolarization.

Second, engineering of a lung-specific hydrogel scaffold to guide branching formation. One interesting approach would be to engineer culture environments resembling the organ native niche by use of scaffold materials that could facilitate the generation of lung organoids that closely recapitulate lung structures and functions. Such methodology has been recently successfully applied to intestinal organoids by placing human stem cells inside a microfluidic chip that was laser-sculpted to resemble the gut (Nikolaev et al., 2020). Remarkably, intestinal stem cells grew and self-organized along a tube-shaped hydrogel scaffold containing proteins found in the gut's extracellular matrix (ECM). Such technology could be translated to lung biology by, for example, engineering of self-guiding “branching” organoids within hydrogel containing lung ECM. Moreover, such technology could allow the coordinated delivery of crucial morphogens into the lung organoids that would facilitate the activation of signaling pathways needed for branching formation in a time-dependent manner.

Third, establishment of a vascularized lung organoid model to facilitate cell differentiation and branching morphogenesis *in vitro*. Vascularization of lung organoids has so far only been established by transplantation of organoids into host animals to promote cell differentiation and maturation (Dye et al., 2016; Chen et al., 2017). Nevertheless, Wimmer and colleagues have recently developed blood vessel organoids derived from hPSC. Notably, these organoids formed capillary networks containing both endothelial cells and pericytes (Wimmer et al., 2019). In another study, partial vascularization of human brain organoids was possible by addition of hPSC-derived endothelial cells from the same patient to re-embedded brain organoids (Pham et al., 2018). Moreover, in a recent study, tumor and neural organoids were vascularized *in vitro* after hPSC-derived human mesodermal progenitors were introduced into the culture (Wörsdörfer et al., 2019). Therefore, vascularized lung organoids could be developed to promote proximodistal cell specification and facilitate visualization of these events *in vitro*. For instance, the use of hPSC-derived mesodermal and lung progenitors may hold the key for the establishment of a vascular system within human lung organoids that could allow analysis of all the stages of branching morphogenesis, including septation and alveolar formation.

## Conclusions

In summary, although there are several valuable lung organoid models available to study different features of lung development, the ultimate goal would be the development of multicellular, highly differentiated, branched organoid systems that more closely resemble the lung architecture. Such complex branching organoid models in combination with the latest technology such as single-cell RNA-sequencing and genome editing tools could prompt the identification of novel molecular cues and cellular interactions occurring during lung bud elongation, branching and alveolarization, providing exciting new insights into branching morphogenesis.

## Author Contributions

AV-A designed and wrote the manuscript. SH and AV-A contributed to the interpretation and discussion, read, and approved the final version of the manuscript.

## Conflict of Interest

The authors declare that the research was conducted in the absence of any commercial or financial relationships that could be construed as a potential conflict of interest.

## Abbreviations

3D: Three-dimensional
ES: embryonic stem
iPSC: induced pluripotent stem cells
hPSC: human pluripotent stem cells
AFE: anterior foregut endoderm
VAFE: ventral anterior foregut endoderm
NKX2.1: NK2 homeobox 1
FGFs: fibroblast growth factors
TGF: transforming growth factor
HH: hedgehog
RA: retinoic acid
CPM: carboxypeptidase M
AQP5: aquaporin 5
AECI: alveolar epithelial cells type 1
SFTPC: surfactant Protein C
AECII: alveolar epithelial cells type 2
GFP: green fluorescent protein
SOX2: SRY-box transcription factor 2
SOX9: SRY-box transcription factor 9
ID2: inhibitor of DNA binding 2
KRT: keratin
NGFR: nerve growth factor receptor
BMP: bone morphogenetic proteins
CRISPR: clustered regularly interspaced short palindromic repeats
Cas9: CRISPR associated protein 9
Grhl2: grainyhead like transcription factor 2
LBOs: lung bud organoids
MUC: Mucin
SCGB3A2: secretoglobin family 3A member 2
SFTPB: surfactant Protein B
HLO: human lung organoids
HOPX: homeodomain-only protein homeobox
PLG: poly(lactide-co-glycolide)
PLO: patterned lung organoids
SCGB1A1: secretoglobin family 1A member 1
BASCs: murine bronchioalveolar stem cells
TSP1: thrombospondin-1
EpCAM: epithelial cell adhesion molecule
SCA-1: stem cell antigen-1
BALO: bronchioalveolar lung organoids
rMC: lung resident mesenchymal cells
PDGFRα: platelet-derived growth factor receptor alpha
αSMA: α-smooth muscle actin
*Apc*: adenomatosis polyposis coli
ECM: extracellular matrix
FOXA2: forkhead-box-protein A2
HUVEC: human umbilical vein endothelial cells
HLF: human lung fibroblasts
MF: mouse fibroblasts
VIM: vimentin
LuMEC: primary mouse lung endothelial cells.
